# Reactogenicity and safety of COVID-19 primary immunisation and booster vaccination regimens: a comparative observational cohort study

**DOI:** 10.1186/s12916-023-02924-5

**Published:** 2023-06-20

**Authors:** Lisette Warkentin, Felix Werner, Nikoletta Zeschick, Thomas Kühlein, Philipp Steininger, Klaus Überla, Isabelle Kaiser, Maria Sebastião, Susann Hueber

**Affiliations:** 1 Institute of General Practice, Friedrich-Alexander-Universität Erlangen-Nürnberg, Uniklinikum Erlangen, Universitätsstraße 29, Erlangen, Germany; 2 Institute of Clinical and Molecular Virology, Friedrich-Alexander-Universität Erlangen-Nürnberg, Uniklinikum Erlangen, Schloßgarten 4, Erlangen, Germany; 3 Department of Medical Informatics, Biometry and Epidemiology, Friedrich-Alexander-Universität Erlangen-Nürnberg, Waldstraße 6, Erlangen, Germany

**Keywords:** COVID-19, Vaccines, Adverse drug reaction, Observational study, Survey, Medical consultation, Reactogenicity

## Abstract

**Background:**

Since the beginning of the COVID-19 vaccination campaigns, recommendations regarding the vaccination have been very dynamic. Although the safety and efficacy of different vaccines have been analysed, data were scarce for vaccine regimens combining different vaccines. We therefore aimed to evaluate and compare the perceived reactogenicity and need for medical consultation after the most frequently applied homologous and heterologous COVID-19 vaccination regimens.

**Methods:**

In an observational cohort study, reactogenicity and safety were assessed within a maximum follow-up time of 124 days using web-based surveys. Reactogenicity was assessed for different vaccination regimens 2 weeks after a vaccination (short-term survey). The following surveys, long-term and follow-up surveys, focused on the utilisation of medical services, including those that were not suspected to be vaccine-related.

**Results:**

Data of 17,269 participants were analysed. The least local reactions were seen after a ChAdOx1 − ChAdOx1 regimen (32.6%, 95% CI [28.2, 37.2]) and the most after the first dose with mRNA-1273 (73.9%, 95% CI [70.5, 77.2]). Systemic reactions were least frequent in participants with a BNT162b2 booster after a homologous primary immunisation with ChAdOx1 (42.9%, 95% CI [32.1, 54.1]) and most frequent after a ChAdOx1 − mRNA-1273 (85.5%, 95% CI [82.9, 87.8]) and mRNA-1273/mRNA-1273 regimen (85.1%, 95% CI [83.2, 87.0]). In the short-term survey, the most common consequences were medication intake and sick leave (after local reactions 0% to 9.9%; after systemic reactions 4.5% to 37.9%). In the long-term and follow-up surveys, between 8.2 and 30.9% of participants reported consulting a doctor and between 0% and 5.4% seeking hospital care. The regression analyses 124 days after the first and after the third dose showed that the odds for reporting medical consultation were comparable between the vaccination regimens.

**Conclusions:**

Our analysis revealed differences in reactogenicity between the COVID-19 vaccines and vaccination regimens in Germany. The lowest reactogenicity as reported by participants was seen with BNT162b2, especially in homologous vaccination regimens. However, in all vaccination regimens reactogenicity rarely led to medical consultations. Small differences in seeking any medical consultation after 6 weeks diminished during the follow-up period. In the end, none of the vaccination regimens was associated with a higher risk for medical consultation.

**Trial registration:**

DRKS DRKS00025881 (https://drks.de/search/de/trial/DRKS00025373). Registered on 14 October 2021. DRKS DRKS00025373 (https://drks.de/search/de/trial/DRKS00025881). Registered on 21 May 2021. Registered retrospectively.

**Supplementary Information:**

The online version contains supplementary material available at 10.1186/s12916-023-02924-5.

## Background

The observation of cowpox inoculation preventing smallpox has been the beginning of vaccinology in the late eighteenth century [1, 2]. Since then many vaccines have been developed, resulting in an enormous impact on global health by reducing the burden of infectious diseases [3]. The World Health Organization (WHO) estimates that vaccines prevented at least ten million deaths between 2010 and 2015 [4]. In 1996 the average time from the preclinical phase to the launch of vaccines was 10 years [5]. Twenty-four years later, on 12 March 2020, the COVID-19 pandemic has been declared by the WHO. The pandemic led to a worldwide interest to develop safe and effective vaccines more rapidly [3]. One year later, by March 2021, already four COVID-19 vaccines have been authorised in Germany: BNT162b2 (BioNTech/Pfizer), mRNA-1273 (Moderna), ChAdOx1 (AstraZeneca) and Ad26.COV2-S (Janssen) [6]. Their safety and efficacy had been demonstrated in large randomized controlled trials [7–10] and many post-authorization trials [11–14].

Since the beginning of COVID-19 vaccination campaigns, recommendations regarding vaccination regimens have been changing frequently. At the beginning of the vaccination campaign, due to characteristics of the study population in phase III studies, ChAdOx1 was not authorised for individuals older than 64 years [15] nor were any of the vaccines authorised for children [16]. Age and gender-specific safety signals [17, 18] and waning of immunity after several months [14] led to adaptions of the recommendations regarding the second dose and booster vaccinations [19]. Recommendations also differed by country: In Germany and France, a single dose of COVID-19 vaccine in individuals with a previous infection has been recommended for primary immunisation (PI), whereas, i.e., in the USA, two doses were recommended [20]. These recommendations led to the administration of a range of vaccination regimens for PI and booster vaccination.

Although the safety of the vaccines and homologous vaccination regimens had been analysed, data on safety and efficacy of vaccination regimens combining different vaccines were scarce. The CoVaKo project (Corona Vakzin Konsortium) analyses different aspects of the efficacy and safety of COVID-19 vaccines. In the CoVaKo safety study, we monitored reactogenicity and safety after COVID-19 vaccinations compared to other vaccinations like influenza or pneumococcal vaccination. In the analysis presented here, we compared the reactogenicity and health problems up to 124 days after the first, second, and third dose of several regimens of COVID-19 vaccination.

## Methods

An observational cohort study was conducted to assess reactogenicity and health problems occurring after different regimens of COVID-19 vaccinations. The reporting of the study is based on the STROBE (Strengthening the Reporting of Observational studies in Epidemiology) recommendations (see Additional file 1: STROBE checklist) [21]).

### Study design and setting

**Fig. 1:**
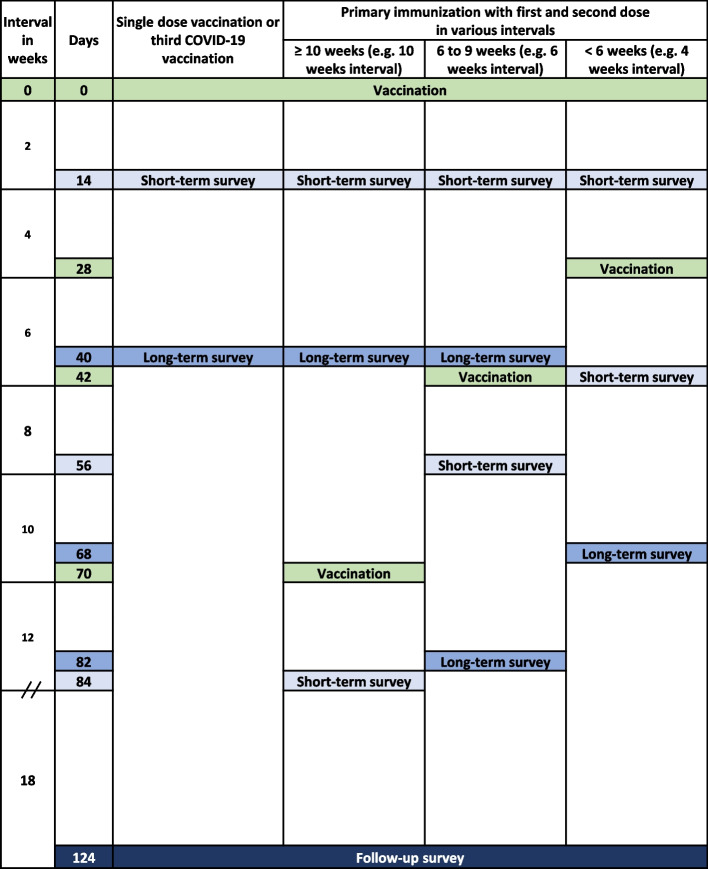
Times of observations. After a COVID-19 vaccination, participants received a short-term survey 14 days after the vaccination and a long-term survey 40 days after the vaccination. Additionally, everyone received a follow-up survey 124 days after the vaccination. If the interval between the two doses was less than 6 weeks, the participant did not receive the first long-term survey to avoid confusion by receiving two surveys in a short period regarding two different vaccinations. If the interval between the two doses was 10 weeks or longer, they did not receive the second long-term survey for the same reason. If the follow-up survey was sent 40 days after the second vaccination, this survey represents the second long-term as well as the follow-up survey

We conducted a longitudinal online survey with focus on reactogenicity and health problems occurring within 124 days after vaccination, leading to medical consultation, medication intake, or sick leave. In a first step, we recruited participants during the process of PI. According to the vaccination recommendations some persons received only one vaccine dose for their PI. With changing vaccination recommendations, we then also recruited participants receiving a third dose (booster) of the COVID-19 vaccine. Participants should preferably register at the time of the first or third vaccination. However, registration was possible during the entire observation period. After registration, participants received links to online surveys at predefined dates. The number of surveys planned varied depending on the number of vaccine doses received and on the time of registration (Fig. 1):

- Participant registered after a single-dose PI or after the third COVID-19 vaccination: 14 days (short-term survey), 40 days (long-term survey), and 124 days (follow-up survey) after vaccination.
- Participant registered after the first dose of a two-dose PI: 14 days (short-term survey) and 40 days (long-term survey) after the first and second vaccination as well as 124 (follow-up survey) days after the first vaccination.
- Participant registered after the second dose of a two-dose PI: 14 days (short-term survey) and 40 days (long-term survey) after the second vaccination as well as 124 (follow-up survey) days after the first vaccination.

Recruitment strategy and surveys had been evaluated in a feasibility study [22] (registered at DRKS: ID DRKS00025881 [23], main study registered at DRKS: ID DRKS00025373 [24]). Recruitment of vaccinated participants commenced on April 17, 2021 (feasibility study), and May 20, 2021 (main study) respectively, in vaccination centres, primary care and company physician practices in Bavaria, Germany. Registration was open until April 17, 2022. Data collection ended on August 28, 2022. Due to the dynamic changes of the vaccination recommendations and the importance of generating real-world evidence on the safety of the various vaccines, we included both, the data of the feasibility study and the main study in this interim analysis. With only minor changes to the survey between the feasibility study and the main study, this approach was considered methodologically valid.

After a COVID-19 vaccination, individuals received a leaflet with information on the study. They could register and give their written informed consent on a web-based platform within 124 days after the vaccination (first or third dose). Inclusion criteria for the safety study were age older than eleven years (in the beginning > 17 years due to recommendations) and a vaccination (against COVID-19, influenza, pneumococcus, tickborne encephalitis, tetanus/diphtheria vaccination with or without pertussis/poliomyelitis, and/or herpes zoster) within the last 124 days. Exclusion criteria were: incomplete registrations, registration before the vaccination date or later than 124 days after vaccination of the first or single dose, and an interval between the first and second dose of less than 14 days. For the study reported here, we analysed only data of participants after COVID-19 vaccination.

### Sample size calculation

An event probability of 0.1% for rare events was assumed. The corresponding 95% confidence interval according to Clopper and Pearson ranges from 0.02 to 0.29% for *N* = 3000. For larger event probabilities, the width of the confidence interval decreases.

### Surveys

The registration form assessed sociodemographic information (age, gender, education, domicile), comorbidities, and information on previous vaccines. Questions on morbidity were based on a modified German version of the Self-Administered Comorbidity Questionnaire (mSCQ-D) [25, 26]. The registration form asked about information on the vaccination, including brand name and batch number. After registration, participants received URL links to the online surveys via email. After receiving a link, participants could respond within five days.

The short-term survey assessed solicited and unsolicited local and systemic reactions. Solicited reactions were reactions after vaccinations like local pain, headache, and fever. The composite outcome ‘local reactions’ contains pain, erythema, swelling, mobility restriction, and abscess. The outcome ‘systemic reactions’ includes headache, fatigue, nausea or vomiting, fever or chills, muscle or joint pain, allergic reactions, dyspnoea, syncope, seizure, dizziness, numbness or paraesthesia, and coagulation disorder. Participants were able to report unsolicited reactions in an open text field. Additionally, the survey asked for possible consequences like medical consultation, medication intake, or sick leave for each symptom. The long-term and follow-up survey focussed only on health problems leading to a doctor's consultation or to seek hospital care, including hospitalisation. Participants were asked about all health problems, not only those suspected to be associated with the vaccination. They could select health problems from a predefined list and/or could report additional health problems in an open text field. Participants were asked to rate if they suspected an association to the vaccination and if the health problem was pre-existing. The surveys can be found in the Additional file 2. If changes had to be made, mainly due to changes in the recommendation, they are indicated in the document.

**Fig. 2:**
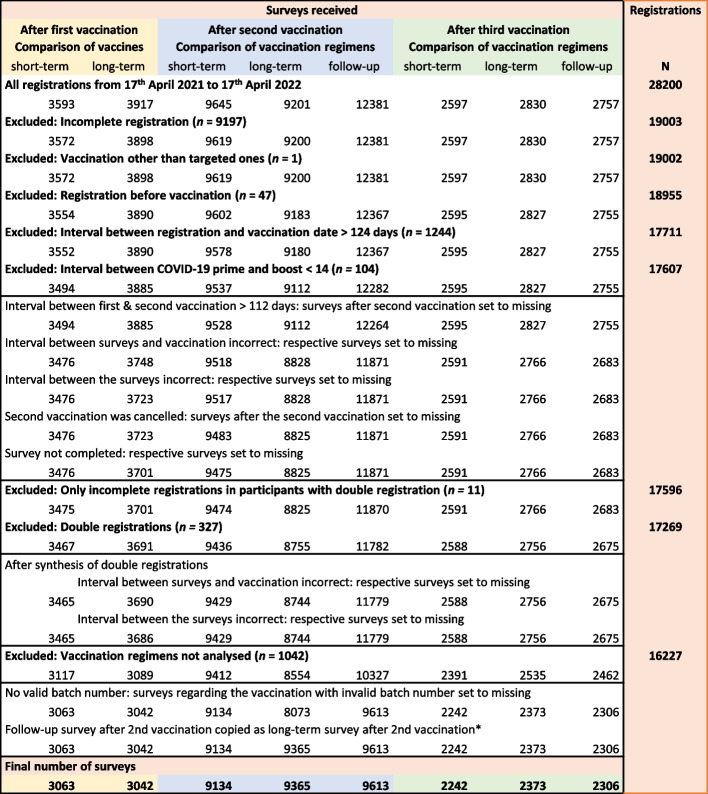
Data selection and preparation process. In case a person registered twice with the same email address, the datasets were synthesised. If one email address was used by several persons, datasets were considered separately. Batch numbers were checked for plausibility. Surveys regarding vaccinations with invalid batch numbers were set to missing. Invalid was defined as a number not known or an incorrect combination of number and vaccine. *If the follow-up survey has been sent 40 days after the second vaccination, this survey represents the second long-term as well as the follow-up survey, as they did not receive a separate long-term survey

For the comparison of the first dose, we included participants receiving one of the four vaccines authorised in Germany by March 2021 [6]. For the second dose and booster, we compared the most common vaccination regimens. The data selection and preparation process are depicted in Fig. 2. The following cohorts were compared:

- First dose: BNT162b2; mRNA-1273; ChAdOx1; Ad26.COV2-S
- Second dose regimens: homologous: BNT162b2 − BNT162b2; mRNA-1273 − mRNA-1273; ChAdOx1 − ChAdOx1; heterologous: ChAdOx1 − BNT162b2; ChAdOx1 − mRNA-1273
- Booster regimens: PI with BNT162b2 or mRNA-1273 (PI mRNA) + BNT162b2; PI mRNA + mRNA-1273; PI with ChAdOx1 (PI vector) + BNT162b2; PI vector + mRNA-1273; PI with BNT162b2 or mRNA-1273 and ChAdOx1 (PI mRNA/vector) + BNT162b2; PI mRNA/vector + mRNA-1273

### Statistical analysis

As age was reported as the year of birth, it was calculated as the difference between the year of registration (2021/2022) and the year of birth provided. In case of implausible age (year of birth before 1900; *n* = 1), weight (lower than 30 kg or higher than 300 kg; *n* = 9), and/or height (lower than 100 cm or higher than 250 cm; *n* = 14) the respective variables were set to missing. Sociodemographic characteristics, comorbidities, and the interval between the first and second dose are reported as a proportion or as a mean/median. Comorbidity in the form of mSCQ-D was calculated. The consequences of reactions on the vaccines were queried in a multiple-choice question. In the descriptive analysis, they were ordered hierarchically. The consequence perceived as most serious is reported in the results (from no consequence to medication intake, sick leave, ambulatory consultation, hospital outpatient consultation, and hospitalisation). Health problems are reported as absolute and relative frequencies. For group comparisons, we performed multivariable logistic regression analyses for the following outcomes: For the short-term surveys the outcomes were local or systemic reactions, for the long- and follow-up surveys doctor’s consultation or hospital care, including hospitalisation (occurred and/or planned, respectively). The models were adjusted for age, gender, mSCQ and interval between vaccination and registration (in days). The regression models for the long-term and follow-up surveys after the second dose were additionally adjusted for the interval between the vaccinations. A first dose with BNT162b2, a vaccination regimen with BNT162b2-BNT162b2 or a PI with mRNA vaccines followed by a BNT162b2 booster represented the reference. In an additional analysis, we examined the influence of a SARS-CoV-2 infection after the vaccination and/or after the last survey on the reporting of the outcomes.

Data were collected using the web-based software platform REDCap (Research Electronic Data Capture), hosted at Uniklinikum Erlangen [27, 28]. Data preparation, analyses, and figures were performed using R Statistical Software (version 4.2.1, R Foundation for Statistical Computing, Vienna, Austria).

## Results

### Sociodemographic characteristics

In total, 14,439 short-term surveys, 14,780 long-term surveys, and 11,919 follow-up surveys have been included in the analysis. Vaccinations with mRNA vaccines were most common. Vaccination groups differed regarding gender and age. Male participants received vector-based vaccines more frequently. More female participants were in the homologous mRNA and heterologous PI regimens. After a heterologous PI, female participants received BNT162b2 and male participants mRNA-1273 more often. Participants receiving only ChAdOx1 had a higher mean age than those receiving mRNA vaccines or Ad26.COV2-S. Regarding the third dose, participants receiving mRNA-1273 were older than those receiving BNT162b2 (except after a homologous vector-based PI). The Ad26.COV2-S cohorts had the highest rates of participants with no pre-existing morbidity (60.0% and 61.0%, respectively), and the homologous ChAdOx1 regimen cohort had the lowest rate (29.6%, 29.9% and 30.0%). The body mass index was comparable between the groups. The interval between the first and second dose was shorter in homologous regimens with mRNA vaccines compared to homologous regimens with ChAdOx1 or heterologous regimens (Table 1).

**Table 1:** Sociodemographic characteristics of participants who responded to the surveys

	**After first vaccination** **Comparison of vaccines**				**After second vaccination** **Comparison of vaccination regimens**					**After third vaccination** **Comparison of vaccination regimens**					
	**mRNA**		**Vector**		**Homologous mRNA**		**Homologous vector**	**Heterologous**		**Homologous PI mRNA**		**Homologous PI vector**		**Heterologous PI mRNA/vector**	
**Short-term survey**															
	BNT	m1273	ChAd	Ad26	BNT- BNT	m1273 m1273	ChAd- ChAd	ChAd-BNT	ChAd-m1273	BNT	m1273	BNT	m1273	BNT	m1273
***N***	**2044**	**687**	**177**	**155**	**5391**	**1386**	**439**	**1098**	**820**	**884**	**782**	**84**	**59**	**204**	**229**
**Gender** (%)															
Female	55.9	62.6	36.7	41.3	57.6	60.2	41.2	59.5	59.8	62.8	56.0	45.2	44.1	60.3	38.9
Male	44.0	37.1	63.3	58.7	42.3	39.5	58.8	40.5	40.2	37.1	44.0	54.8	55.9	39.7	61.1
Divers	0	0.3	0	0	0.1	0.3	0	0	0.1	0.1	0	0	0	0	0
**Age**															
Mean	42.1	41.4	46.1	39.2	44.8	43.4	56.3	48.3	47.5	38.1	47.3	51.9	51.1	40.1	51.9
SD	14.8	14.4	15.9	12.9	15.3	14.4	15.6	13.9	13.9	16.4	11.4	15.8	12.7	14.3	12.9
**No pre-existing diseases** (%)															
	44.8	44.7	48.0	60.0	40.0	42.3	29.6	36.7	42.1	47.3	41.4	36.9	40.7	53.4	48.5
**m-SCQ-D**															
Median	0	0	0	0	0	0	1	0	0	0	0	0	0	0	0
IQR	0–2	0–2	0–2	0–2	0–2	0–2	0–3	0–2	0–2	0–2	0–2	0–2	0–2	0–1	0–2
**BMI**															
Mean	25.4	25.7	25.2	25.1	25.8	25.8	26.9	25.8	25.8	24.7	25.9	25.7	28.7	25.1	26.1
SD	5.4	5.4	4.3	4.0	5.4	5.4	5.2	5.0	5.3	5	5.1	5.1	5.4	5.4	4.6
NA	27	16	3	1	62	22	5	12	8	10	8	1	0	2	2
**Participants with other vaccinations 8 weeks before first/third vaccination** (%)															
	5.8	4.8	7.9	3.2	5.5	5.2	5.5	5.9	7.1	25.7	25.7	41.7	32.2	35.8	33.6
**Time between 1**^**st**^** and 2**^**nd**^** vaccination** (days)															
Mean					40.2	41.6	76.6	68.6	75.9						
SD					5.8	3.2	12.8	13.1	11.4						
NA					62	22	5	12	8						
**Long-term survey**															
	BNT	m1273	ChAd	Ad26	BNT- BNT	m1273 m1273	ChAd- ChAd	ChAd-BNT	ChAd-m1273	mRNA PI-BNT	mRNA PI-m1273	Vector PI-BNT	Vector PI-m1273	Het. PI-BNT	Het. PI-m1273
***N***	**2002**	**689**	**192**	**159**	**5513**	**1378**	**426**	**1201**	**847**	**932**	**829**	**92**	**61**	**226**	**233**
**Gender** (%)															
Female	56.0	61.4	37.0	42.1	58.1	60.7	41.3	61.5	60.9	62.6	56.0	45.7	49.2	63.7	38.6
Male	44.0	38.5	63.0	57.9	41.8	39.0	58.7	38.5	39.0	37.2	44.0	54.3	50.8	36.3	61.4
Divers	0.0	0.1	0.0	0.0	0.1	0.3	0.0	0.0	0.1	0.2	0.0	0.0	0.0	0.0	0.0
**Age**															
Mean	44.6	41.7	48.4	39.7	45.1	43.9	57.3	48.5	48.4	38.4	47.6	51.7	50.8	40.3	51.9
SD	15.3	14.4	16.2	12.9	15.3	14.5	15.6	13.5	14.1	16.3	11.3	15.8	13.0	14.1	13.0
**No pre-existing diseases** (%)															
	40.0	44.1	42.2	61.0	39.8	41.4	30.0	37.1	41.8	47.6	41.4	37.0	44.3	52.2	46.8
**m-SCQ-D**															
Median	0.0	0.0	0.0	0.0	0.0	0.0	0.0	0.0	0.0	0.0	0.0	0.0	0.0	0.0	0.0
IQR	0–2	0–2	0–2	0–2	0–2	0–2	0–3	0–2	0–1	0–2	0–2	0–2	0–2	0–1	0–1,5
**BMI**															
Mean	25.8	25.8	25.6	25.2	25.8	25.8	26.6	25.8	25.8	24.9	26.0	26.0	28.4	25.2	26.3
SD	5.6	5.4	4.6	4.2	5.4	5.4	5.2	5.1	5.3	5.2	5.2	5.1	5.3	5.6	4.9
NA	28	13	2	1	64	22	5	10	7	10	8	1	0	3	2
**Participants with other vaccinations 8 weeks before first vaccination** (%)															
	5.7	4.6	7.3	3.1	5.5	5.3	4.9	6.2	7.3	25.4	26.7	42.4	27.9	35.8	32.2
**Time between 1**^**st**^** and 2**^**nd**^** vaccination** (days)															
Mean					40.2	41.5	76.6	68.6	75.9						
SD					5.8	3.2	12.8	13.1	11.4						
NA					7	1	0	1	0						
**Follow-up survey**															
	BNT	m1273	ChAd	Ad26	BNT- BNT	m1273 m1273	ChAd- ChAd	ChAd-BNT	ChAd-m1273	mRNA PI-BNT	mRNA PI-m1273	Vector PI-BNT	Vector PI-m1273	Het. PI-BNT	Het. PI-m1273
***N***					**5539**	**1342**	**471**	**1382**	**879**	**916**	**796**	**94**	**60**	**207**	**233**
**Gender** (%)															
Female					58.8	61.2	41.0	61.9	60.4	63.3	55.8	47.9	46.7	66.7	38.6
Male					41.1	38.5	59.0	38.1	39.5	36.6	44.2	52.1	52.3	33.3	61.4
Divers					0.1	0.4	0.0	0.0	0.1	0.2	0.0	0.0	0.0	0.0	0.0
**Age**															
Mean					45.6	44.6	57.3	48.9	48.4	39.0	47.8	52.0	50.8	40.9	51.9
SD					15.2	14.3	15.4	13.6	14.3	16.6	11.4	15.9	12.6	14.0	13.0
**No pre-existing diseases** (%)															
					38.9	39.9	29.9	37.4	41.9	46.8	41.2	36.2	43.3	50.7	45.9
**m-SCQ-D**															
Median					0	0	0	0	0	0	0	0	0	0	0
IQR					0–2	0–2	0–3	0–2	0–2	0–2	0–2	0–2	0–2	0–2	0–2
**BMI**															
Mean					25.8	25.9	26.8	25.6	25.9	24.9	26.0	25.7	28.2	25.0	26.2
SD					5.4	5.4	5.3	4.9	5.2	5.2	5.1	5.3	5.2	5.2	4.9
NA					59	21	6	14	1	10	8	1	0	3	2
**Participants with other vaccinations 8 weeks before first vaccination** (%)															
					7.2	4.8	5.1	6.2	7.3	26.3	26.6	40.4	26.7	37.2	33.5
**Time between 1**^**st**^** and 2**^**nd**^** vaccination** (days)															
Mean					40.2	41.6	76.8	70.5	77.8						
SD					6	3.8	13	12.9	10.9						
NA					6	0	0	0	0						

*PI* primary immunisation, *BNT* BNT126b2, *m1273* mRNA-1273, *ChAd* ChAdOx1, *Ad26* Ad26.COV2-S, *PI mRNA* PI with BNT162b2 or mRNA-1273, *PI vector* PI with ChAdOx1, *PI mRNA/vector* PI with BNT162b2 or mRNA-1273 and ChAdOx1, *m-SCQ-D* modified German version of the Self-Administered Comorbidity Questionnaire, *NA* not applicable

### Descriptive results of the short-term survey

#### First vaccination

Local reactions were most common after a vaccination with an mRNA vaccine, whereas systemic reactions were more frequent after vector-based vaccines (local: BNT162b2: 57.5%, 95% CI [55.3, 59.6]; mRNA-1273: 73.9%, 95% CI [70.5, 77.2]; ChAdOx1: 54.2%, 95% CI [46.6, 61.7]; Ad26.COV2-S: 47.7%, 95% CI [39.7, 55.9]; systemic: BNT162b2: 48.5%, 95% CI [46.3, 50.7]; mRNA-1273: 59.2%, 95% CI [55.5, 62.9]; ChAdOx1: 81.9%, 95% CI [75.5, 87.3]; Ad26.COV2-S: 77.4%, 95% CI [70, 83.7]). More participants in the BNT162b2 or ChAdOx1 cohort reported that local reactions were without consequences than in the mRNA-1273 or Ad26.COV2-S cohort (BNT162b2: 95.0%, 95% CI [93.6, 96.2]; ChAdOx1 94.8%, 95% CI [88.3, 98.3]; mRNA-1273 86.2%, 95% CI [82.9, 89.1]; Ad26.COV2-S 90.5%, 95% CI [81.5, 96.1]). No consequences of systemic reactions were more frequently reported after mRNA vaccines (BNT162b2: 71.3%, 95% CI [68.3, 74.1]; mRNA-1273: 67.1%, 95% CI [62.3, 71.6]; ChAdOx1: 46.2%, 95% CI [37.9, 54.7]; Ad26.COV2-S: 40.8%, 95% CI [32, 50.2]).

#### Second vaccination

Participants with a ChAdOx1 vaccination reported fewer local reactions than those with an mRNA vaccination (ChAdOx1: 32.6%, 95% CI [28.2, 37.2]; BNT162b2: 55.8%, 95% CI [54.4, 57.1] (homologous); 61.6%, 95% CI [58.6, 64.5] (heterologous); mRNA-1273: 78.7%, 95% CI [76.5, 80.8] (homologous); 77.2%, 95% CI [74.2, 80.0] (heterologous)). Systemic reactions were least frequent after a ChAdOx1 vaccination (ChAdOx1: 48.5%, 95% CI [43.8, 53.3]; BNT162b2: 61.8%, 95% CI [60.4, 63.1] (homologous); 76.0%, 95% CI [73.3, 78.5] (heterologous); mRNA-1273: 85.1%, 95% CI [83.2, 87.0] (homologous); 85.5%, 95% CI [82.9, 87.8] (heterologous)). No consequences of systemic reactions were most frequently reported after homologous BNT162b2 or ChAdOx1 vaccination (BNT162b2: 58.6%, 95% CI [56.9, 60.3]; ChAdOx1: 58.2%, 95% CI [51.3, 64.9]).

#### Third vaccination

**Fig. 3:**
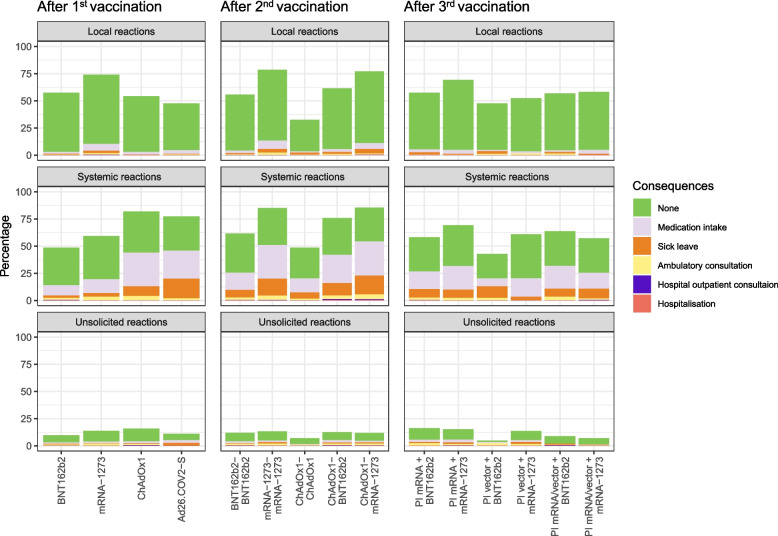
Reactions after a first, second or third COVID-19 vaccination with different regimens. Local, systemic, and unsolicited reactions 14 days after the first, second, and third COVID-19 vaccination in participants with different vaccination regimens with consequences in hierarchical order, as multiple choice was possible. The consequence perceived as most serious is reported (from no consequence to medication intake, sick leave, ambulatory consultation, hospital outpatient consultation, and hospitalisation). Bars show the percentage of participants who reported at least one reaction in the respective category. Participants’ characteristics of each vaccine or vaccination regimen group are depicted in Table 1. PI mRNA = PI with BNT162b2 or mRNA-1273. PI vector: PI with ChAdOx1. PI mRNA/vector: PI with BNT162b2 or mRNA-1273 and ChAdOx1

Most reactions were reported after receiving an mRNA-1273 booster after a PI with mRNA vaccines (local: 69.2%, 95% CI [65.8, 72.4]; systemic: 69.3%, 95% CI [65.9, 72.5]). The least reactions were reported after receiving a BNT162b2 vaccination after a PI with vector-based vaccines (local: 47.6%, 95% CI [36.6, 58.8]; systemic: 42.9%, 95% CI [32.1, 54.1]). As compared to the first and second vaccination, differences between groups were smaller after the third vaccination (Fig. 3 and Additional file 3: Table S1).

In the short-term surveys, between 0.0 and 1.7% of participants reported that they had a SARS-CoV-2 infection since the respective vaccination (Additional file 3: Table S1).

### Descriptive results of the long-term and follow-up surveys

In the long-term survey, 13.0% to 16.7% sought medical consultation (not necessarily associated with the vaccination) after the first vaccination, 14.8% to 16.8% after the second and 9.8% to 20.7% after the third vaccination. In the follow-up surveys, 16.4% to 21.6% reported any medical consultation after the primary immunisation and 26.7% to 32.3% after the third vaccination. A small percentage (0% -5%) reported seeking hospital care. Up to 9% of the participants with medical consultation reported that all of the health problems that led to medical consultation were unknown and that they suspected an association with the vaccination. In the follow-up survey after the third dose, between 22.2 and 26.0% of participants reported that they had a SARS-CoV-2 infection since the last survey. In the follow-up survey after the second dose and in the long-term survey less than 2.5% reported a SARS-CoV-2 infection (Table 2).

**Table 2:** Descriptive results of the long-term and follow surveys

	**After first vaccination** **Comparison of vaccines**				**After second vaccination** **Comparison of vaccination regimens**										
	**mRNA**		**Vector**		**Homologous mRNA**				**Homologous vector**			**Heterologous**			
	BNT	1273	ChAd	Ad26	BNT - BNT		1273 – 1273		ChAd - ChAd			ChAd - BNT		ChAd - 1273	
	LT	LT	LT	LT	LT	FU	LT	FU	LT	FU		LT	FU	LT	FU
***N***	**2002**	**689**	**192**	**159**	**5513**	**5539**	**1378**	**1342**	**426**	**471**		**1201**	**1382**	**847**	**879**
**Participants with SARS-CoV-2 infection after vaccination** (%)															
	0.6	0.0	0.5	1.3	0.5	1.3	0.4	0.7	0.0	0.0		0.1	0.2	0.0	0.0
NA (n)	9	2	1	1	14	1	1	0	0	1		6	6	4	4
**Medical consultation** (%)															
Doctor’s consultation	11.0	11.2	15.1	12.0	12.8	19.4	13.7	18.3	13.4	15.9		14.0	15.8	13.3	14.0
Doctor’s consultation in planning	1.8	2.5	1.0	4.4	2.4	2.6	2.7	3.0	1.2	2.1		2.6	2.5	2.2	2.2
Hospital care	1.6	1.3	2.6	0.6	1.7	2.5	1.1	2.2	2.6	2.5		1.1	1.1	0.7	1.1
Hospital care in planning	0.0	0.1	0.0	1.3	0.1	0.4	0.4	0.3	0.2	0.2		0.1	0.4	0.0	0.0
**Participants with at least one medical consultation**															
%	13.0	13.8	16.7	16.5	15.4	21.4	16.5	21.6	14.8	18.3		16.8	18.4	15.7	16.4
*** n***	**261**	**95**	**32**	**26**	**849**	**1183**	**227**	**290**	**63**	**86**		**203**	**254**	**133**	**144**
**Thereof:**															
**HPlMC was unknown to the participant** (%)															
All HPlMC	48.7	45.3	68.8	46.2	51.0	51.8	57.3	54.1	50.8	43.0		53.7	49.2	56.4	56.9
At least one	35.2	35.8	31.2	38.5	35.9	34.5	35.7	32.4	33.3	36.0		30.0	34.3	31.6	28.5
**Participant suspected association of HPlMC to vaccination** (%)															
Regarding all HPlMC	5.0	12.6	3.1	7.7	4.6	2.9	6.6	3.8	1.6	3.5		4.9	2.8	5.3	4.2
Regarding at least on HPlMC	23.0	17.9	18.8	23.1	21.9	17.7	30.8	19.7	17.5	10.5		16.7	16.1	18.8	18.1
**All HPlMC unknown and association with vaccination is suspected by the participant** (%)															
	4.2	9.4	3.1	7.6	3.9	2.4	5.7	2.8	1.6	2.3		4.4	2.4	5.3	4.2
**Health problems leading to medical consultation** (%)															
Musculoskeletal disorders	36.8	49.5	34.3	41.9	39.9	40.3	43.5	38.3	49.1	48.7		38.4	38.9	38.3	38.2
General symptoms	42.5	52.6	31.2	38.1	42.8	41.9	57.2	46.9	34.8	29.0		33.0	31.5	33.1	36.1
Neurological disorders	36.4	44.2	37.4	34.3	32.4	29.2	43.1	30.7	39.6	26.7		31.5	26.0	34.6	29.8
Cardiovascular disorders	19.2	9.5	18.7	15.2	17.7	15.7	15.0	14.1	17.4	16.2		8.4	10.2	17.3	13.2
Unsolicited health problems	52.5	55.8	56.1	45.7	51.6	48.9	45.7	48.0	53.8	54.5		53.2	46.8	57.9	56.9
No health problem named	6.5	6.3	6.2	3.8	5.4	4.7	19.4	4.1	3.2	3.5		6.4	6.7	4.5	4.2
		**After third vaccination: Comparison of vaccination regimens**													
		**Homologous primary immunisation mRNA**				**Homologous primary immunisation vector**					**Heterologous primary immunisation mRNA/vector**				
		BNT		1273		BNT		1273			BNT			1273	
		LT	FU	LT	FU	LT	FU	LT	FU		LT		FU	LT	FU
***N***		**932**	**916**	**829**	**796**	**92**	**94**	**61**	**60**		**226**		**207**	**233**	**233**
**Participants with COVID-19 infection after vaccination** (%, no missing values)															
		2.4	26.0	1.9	22.2	1.1	23.4	0.0	23.3		1.8		24.6	1.7	24.5
**Medical consultation** (%)															
Doctor’s consultation		13.4	29.9	13.6	29.8	18.5	30.9	8.2	25.0		11.9		26.6	12.4	24.9
Doctor’s consultation in planning		3.8	1.6	2.5	2.0	2.2	0.0	1.6	1.7		1.8		0.5	2.1	2.1
Hospital care		1.3	2.7	1.6	2.3	5.4	4.3	0.0	3.3		1.8		3.9	0.9	2.1
Hospital care in planning		0.3	0.1	0.4	0.1	0.0	0.0	0.0	0.0		0.0		0.5	0.0	0.0
**Participants with at least one medical consultation**															
%		17.4	31.7	16.9	32.3	20.7	31.9	9.8	26.7		13.7		29.5	14.6	27.5
*** n***		**162**	**290**	**140**	**257**	**19**	**30**	**6**	**16**		**31**		**61**	**34**	**64**
**Thereof:**															
**HPiMC was unknown to the participant** (%)															
All HPlMC		48.1	50.3	55.0	56.0	52.6	70.0	33.3	43.8		51.6		55.7	47.1	60.9
At least one		34.6	36.2	32.9	33.9	31.6	20.0	16.7	25.0		32.3		29.5	26.5	28.1
**Participant suspected association of HPlMC to vaccination** (%)															
Regarding all HPlMC		2.5	2.4	0.0	0.8	0.0	0.0	0.0	0.0		6.5		1.6	2.9	0.0
Regarding at least on HPlMC		16.7	10.3	18.6	14.8	10.5	13.3	33.3	6.2		12.9		6.6	2.9	0.0
**All HPlMC unknown and association with vaccination is suspected by the participant** (%)															
		1.8	2.1	0.0	0.8	0.0	0.0	0.0	0.0		6.5		1.6	2.9	0.0
**Health problems leading to medical consultation** (%)															
Musculoskeletal disorders		35.8	41.7	36.4	35.8	26.3	33.3	33.5	25.0		38.8		44.2	26.5	29.7
General symptoms		40.1	48.9	40.7	54.5	36.8	43.4	50.2	43.7		42.0		52.4	38.2	56.2
Neurological disorders		25.9	31.0	33.5	32.7	21.0	30.0	33.5	31.2		35.5		29.5	23.5	25.0
Cardiovascular disorders		21.6	16.5	15.7	18.3	5.3	10.0	16.7	6.2		12.9		13.1	8.8	6.2
Unsolicited health problems		47.5	39.3	43.5	48.6	47.3	53.4	66.9	56.2		51.7		49.1	47.0	39.0
No health problem named		3.1	6.2	7.9	2.7	5.3	0.0	0.0	6.2		6.5		1.6	5.9	3.1

*BNT* BNT126b2, *1273* mRNA-1273, *ChAd* ChAdOx1, *Ad26* Ad26.COV2-S, *mRNA* BNT162b2 or mRNA-1273, *vector* ChAdOx1, *mRNA/vector* BNT162b2 or mRNA-1273 and ChAdOx1, *LT* long-term survey, *FU* follow-up survey, *HPlMC* health problem leading to medical consultation

### Multivariate regression analyses of local and systemic reactions reported in the short-term surveys

#### First vaccination

Compared to BNT162b2, mRNA-1273 was associated with a higher risk of local reactions (OR = 2.05, 95% CI [1.69, 2.51]), Ad26.COV2-S with a lower risk (OR = 0.68, 95% CI [0.49, 0.96]), and ChAdOx1 with an equal risk (OR 1.10, 95% CI [0.80, 1.53]). Higher age was associated with less reported reactions (OR = 0.97, 95% CI [0.97, 0.98]), whereas female gender was associated with more reported local reactions (OR = 2.09, 95% CI [1.79, 2.44]). The mSCQ (*p* = 0.0878) and the interval between vaccination and registration (*p* = 0.3121) had no impact on local reactions.

Regarding systemic reactions, logistic regression showed the highest effects in ChAdOx1 (OR = 6.41, 95% CI [4.31, 9.80]), followed by Ad26.COV2-S (OR = 4.21, 95% CI [2.86, 6.36]) and mRNA-1273 (OR = 1.48, 95% CI [1.24, 1.78]). Higher age was associated with less reported reactions (OR = 0.97, 95% CI [0.97, 0.98]), whereas female gender (OR = 1.80, 95% CI [1.54, 2.10]), higher mSCQ (OR = 1.19, 95% CI [1.13, 1.25]), and higher vaccination-registration interval (OR = 1.03, 95% CI [1.01, 1.05]) were associated with a higher risk of reporting systemic reactions.

#### Second vaccination

An mRNA-1273 s dose was associated with a higher risk of local reactions in a homologous (OR = 3.10, 95% CI [2.68, 3.59]) as well as a heterologous regimen (3.18, 95% CI [2.40, 4.22]) compared to a homologous BNT162b2 regimen. A second dose of BNT162b2 in a heterologous regimen was associated with a higher symptom rate of local reactions than in a homologous regimen (OR = 1.49, 95% CI [1.19, 1.88]). Least frequent local reactions were seen in a homologous regimen with ChAdOx1 (OR = 0.57, 95% CI [0.42, 0.76]). Regarding systemic reactions, associations remain the same for mRNA-1273 and BNT162b2 second dose, whereas the effects are higher than for local reactions. A homologous ChAdOx1 regimen was not associated with more systemic reactions (*p* = 0.4262). Higher age was associated with less local and systemic reactions. Female age, higher mSCQ and larger interval between vaccination and registration were associated with a higher risk of local or systemic reactions. The interval between the vaccinations had no statistically significant impact on the reporting of local reactions (*p* = 0.379); however, a larger vaccination-registration interval was associated with less reported systemic reactions (Table 3).

**Table 3:** Multivariate regression analyses of local and systemic reactions reported in the short-term surveys

	**Local reactions 14** ** days after the vaccination**				**Systemic reactions 14** ** days after the vaccination**			
	**OR**	**95% CI**		***p-value***	**OR**	**95% CI**		***p-value***
		LL	UL			LL	UL	
**First vaccination (*n*** ** = 3060, reference = BNT162b2)**								
Intercept	3.01	2.28	3.85	< 0.0001	1.82	1.40	2.35	< 0.0001
mRNA-1273	2.05	1.69	2.51	< 0.0001	1.48	1.24	1.78	< 0.0001
ChAdOx1	1.10	0.80	1.53	0.5536	6.41	4.31	9.80	< 0.0001
Ad26.COV2-S	0.68	0.49	0.96	0.0278	4.21	2.86	6.36	< 0.0001
Age	0.97	0.97	0.98	< 0.0001	0.97	0.97	0.98	< 0.0001
Female	2.09	1.79	2.44	< 0.0001	1.80	1.54	2.10	< 0.0001
mSCQ	1.05	0.99	1.10	0.0878	1.19	1.13	1.25	< 0.0001
Interval V-R	1.01	0.99	1.04	0.3121	1.03	1.01	1.05	0.0153
**Second vaccination (*n*** ** = 9124, reference = BNT162b2 + BNT162b2)**								
Intercept	3.46	2.65	4.52	< 0.0001	7.05	5.28	9.44	< 0.0001
mRNA-1273 − mRNA-1273	3.10	2.68	3.59	< 0.0001	3.80	3.23	4.48	< 0.0001
ChAdOx1 − ChAdOx1	0.57	0.42	0.76	0.0019	1.13	0.83	1.55	0.4262
ChAdOx1 − BNT162b2	1.49	1.19	1.88	0.0057	2.97	2.31	3.85	< 0.0001
ChAdOx1 − mRNA-1273	3.18	2.40	4.22	< 0.0001	5.57	4.06	7.69	< 0.0001
Age	0.97	0.97	0.97	< 0.0001	0.97	0.96	0.97	< 0.0001
Female	2.12	1.93	2.32	< 0.0001	1.98	1.80	2.18	< 0.0001
mSCQ	1.09	1.06	1.12	< 0.0001	1.12	1.09	1.16	< 0.0001
Interval V-R	1.01	1.00	1.01	< 0.0001	1.01	1.01	1.01	< 0.0001
Interval to 2nd dose	1.00	0.99	1.00	0.379	0.99	0.98	1.00	0.0059
**Third vaccination (*n*** ** = 2241, reference = PI mRNA + BNT162b2)**								
Intercept	2.32	1.72	3.14	< 0.0001	3.37	2.48	4.59	< 0.0001
PI mRNA + mRNA-1273	2.33	1.87	2.91	< 0.0001	2.48	1.98	3.10	< 0.0001
PI vector + BNT162b2	1.08	0.67	1.73	0.7605	0.87	0.54	1.41	0.5810
PI vector + mRNA-1273	1.27	0.73	2.20	0.3991	1.87	1.07	3.34	0.0293
PI mRNA/vector + BNT162b2	1.07	0.78	1.47	0.6678	1.41	1.02	1.96	0.0408
PI mRNA/vector + mRNA-1273	1.82	1.32	2.51	0.0003	1.87	1.36	2.59	0.0001
Age	0.97	0.97	0.98	< 0.0001	0.96	0.96	0.97	< 0.0001
Female	1.98	1.66	2.38	< 0.0001	1.69	1.41	2.03	< 0.0001
mSCQ	1.10	1.04	1.17	0.0015	1.13	1.06	1.20	0.0002
Interval V-R	1.01	0.98	1.04	0.5416	1.05	1.02	1.08	0.0016

*CI* confidence interval, *LL* lower limit, *UL* upper limit, *interval V-R* interval between vaccination and registration to the study, *PI mRNA* PI with BNT162b2 or mRNA-1273, *PI vector* PI with ChAdOx1. *PI mRNA/vector* PI with BNT162b2 or mRNA-1273 and ChAdOx1. Age in years, reference gender: male (divers: excluded due to small n), mSCQ: continuous variable, interval in days. Participants excluded from analysis: gender = divers: first vaccination: *n* = 3, second: *n* = 8, third: *n* = 1; NA for “interval to 2^nd^ dose: second vaccination: *n* = 2)

#### Third vaccination

A third dose of mRNA-1273 after a homologous PI with mRNA vaccines and after a heterologous PI was associated with a higher reporting of local reactions (OR = 2.33, 95% CI [1.87, 2.91] and OR = 1.82, 95% CI [(1.32, 2.51]), however, not after a vector PI (*p* = 0.399).

Regarding systemic reactions, a mRNA-1273 third dose was associated with a higher reporting after all PI regimens (PI mRNA: OR = 2.48, 95% CI [1.98, 3.10], PI vector: OR = 1.87, 95% CI [1.07, 3.34], PI heterologous: OR = 1.87, 95% CI [1.36, 2.59]), as was a BNT162b2 after a heterologous PI (OR = 1.41, 95% CI [1.02, 1.96]). Again, a higher age was associated with less reported local and systemic reaction, whereas female gender and higher mSCQ showed a higher OR. A higher interval between vaccination and registration was associated with a higher frequency of systemic but not local reactions (*p* = 0.5416) (Table 3).

### Multivariate regression analyses of medical consultations reported in the long-term and follow-up surveys

#### Long-term survey — first vaccination

Receiving ChAdOx1 or Ad26.COV2-S was associated with more doctor’s consultations compared to BNT162b2 (OR = 1.65, 95% CI [1.07, 2.47] and OR = 1.70, 95% CI [1.06, 2.64] respectively); no statistically significant association was seen for first dose with mRNA-1273 (*p* = 0.2785). Regarding hospital care, the logistic regression showed no vaccine effect. A higher mSCQ was associated with a higher risk for both doctor’s consultations and seeking hospital care (OR = 1.27, 95% CI [1.20, 1.34] and OR = 1.26, 95% CI [1.12, 1.40]). Regarding doctor’s consultations, higher age was associated with a lower risk (OR = 0.99, 95% CI [0.98, 1.00]) and female gender with a higher risk of reporting (OR = 1.61, 95% CI [1.28, 2.02]) (Table 4).

**Table 4:** Multivariate regression analyses of doctor’s consultations and hospital care reported in the long-term and follow-up surveys

	OR	95% CI		*p-value*	OR	95% CI		*p-value*
		LL	UL			LL	UL	
	**Long-term survey:** **Doctor’s consultation**				**Long-term survey:** **Hospital care**			
**First vaccination (*n*** **= 3038, reference = BNT162b2)**								
Intercept	0.11	0.07	0.16	< 0.0001	0.01	0.00	0.04	< 0.0001
mRNA-1273	1.16	0.89	1.50	0.2785	1.03	0.47	2.07	0.9368
ChAdOx1	1.65	1.07	2.47	0.0189	2.16	0.72	5.28	0.1223
Ad26.COV2-S	1.70	1.06	2.64	0.0214	1.56	0.37	4.55	0.4733
Age	0.99	0.98	1.00	0.0281	0.99	0.97	1.01	0.1709
Female	1.61	1.28	2.02	< 0.0001	1.44	0.81	2.66	0.2248
mSCQ	1.27	1.20	1.34	< 0.0001	1.26	1.12	1.40	< 0.0001
Interval V-R	1.01	1.00	1.01	0.1426	1.02	1.00	1.04	0.0541
**Second vaccination (*n*** **= 9351, reference = BNT162b2 + BNT162b2)**								
Intercept	0.09	0.06	0.13	< 0.0001	0.01	0.00	0.02	< 0.0001
mRNA-1273 + mRNA-1273	1.14	0.97	1.34	0.1164	0.87	0.52	1.39	0.5793
ChAdOx1 + ChAdOx1	0.72	0.48	1.07	0.1130	0.78	0.25	2.17	0.6434
ChAdOx1 + BNT162b2	0.85	0.64	1.13	0.2711	0.39	0.16	0.91	0.0380
ChAdOx1 + mRNA-1273	0.80	0.57	1.12	0.2047	0.23	0.06	0.71	0.0160
Age	1.00	0.99	1.0024	0.4154	1.00	0.99	1.02	0.5247
Female	1.64	1.45	1.85	< 0.0001	0.89	0.64	1.24	0.4714
mSCQ	1.24	1.20	1.28	< 0.0001	1.22	1.14	1.30	< 0.0001
Interval V-R	1.01	1.01	1.01	< 0.0001	1.02	1.01	1.03	0.0005
Interval to 2nd dose	1.00	1.00	1.01	0.2156	1.01	0.99	1.04	0.3703
**Third vaccination (*n*** **= 2371, reference = PI mRNA + BNT162b2)**								
Intercept	0.15	0.10	0.23	< 0.0001	0.01	0.00	0.03	< 0.0001
PI mRNA + mRNA-1273	0.95	0.73	1.24	0.7316	1.12	0.54	2.33	0.7654
PI vector + BNT162b2	1.25	0.70	2.14	0.4362	2.62	0.80	7.32	0.0825
PI vector + mRNA-1273	0.44	0.16	1.01	0.0759	NA*	NA*	NA*	NA*
PI mRNA/vector + BNT162b2	0.77	0.49	1.16	0.2225	1.09	0.31	3.08	0.8780
PI mRNA/vector + mRNA-1273	0.91	0.59	1.39	0.6809	0.46	0.07	1.71	0.3125
Age	1.00	0.99	1.00	0.2941	1.01	0.99	1.03	0.4204
Female	1.17	0.93	1.48	0.1794	0.71	0.38	1.34	0.2852
mSCQ	1.28	1.20	1.36	< 0.0001	1.25	1.08	1.44	0.0018
Interval V-R	1.02	1.01	1.04	0.0052	1.04	1.00	1.07	0.0556
	**Follow-up survey:** **Doctor’s consultation**				**Follow-up survey:** **Hospital care**			
**First/Second vaccination (*n*** **= 9601, reference = BNT162b2 + BNT162b2)**								
Intercept	0.22	0.16	0.30	< 0.0001	0.02	0.01	0.07	< 0.0001
mRNA-1273 + mRNA-1273	1.08	0.92	1.25	0.3450	1.32	0.66	1.42	0.9261
ChAdOx1 + ChAdOx1	0.92	0.65	1.30	0.6490	0.54	0.39	2.16	0.9160
ChAdOx1 + BNT162b2	0.93	0.72	1.20	0.5850	0.71	0.27	1.07	0.0923
ChAdOx1 + mRNA-1273	0.84	0.61	1.15	0.2850	0.80	0.17	1.04	0.0749
Age	1.00	0.99	1.00	0.1550	1.00	0.99	1.01	0.5637
Female	1.38	1.24	1.54	< 0.0001	1.59	0.66	1.12	0.2628
mSCQ	1.26	1.23	1.30	< 0.0001	1.26	1.14	1.28	< 0.0001
Interval V-R	1.01	1.01	1.01	< 0.0001	1.02	1.01	1.02	< 0.0001
Interval to 2nd dose	1.00	0.99	1.00	0.1480	1.00	0.98	1.01	0.7033
**Third vaccination (*n*** **= 2304, reference = PI mRNA + BNT162b2)**								
Intercept	0.34	0.25	0.47	< 0.0001	0.01	0.01	0.03	< 0.0001
PI mRNA + mRNA-1273	1.04	0.84	1.29	0.7214	0.75	0.40	1.38	0.3583
PI vector + BNT162b2	0.95	0.58	1.52	0.8358	1.14	0.32	3.11	0.8163
PI vector + mRNA-1273	0.79	0.42	1.43	0.4563	0.96	0.15	3.40	0.9615
PI mRNA/vector + BNT162b2	0.82	0.58	1.15	0.2562	1.58	0.69	3.32	0.2517
PI mRNA/vector + mRNA-1273	0.87	0.62	1.21	0.4139	0.62	0.21	1.55	0.3498
Age	1.00	0.99	1.00	0.6220	1.02	1.00	1.03	0.0843
Female	1.18	0.98	1.42	0.0811	0.89	0.54	1.49	0.6506
mSCQ	1.19	1.13	1.26	< 0.0001	1.14	1.00	1.28	0.0190
Interval V-R	1.01	1.01	1.02	0.0013	1.01	1.00	1.03	0.0392

*CI* confidence interval, *LL* lower limit, *UL* = upper limit, *interval V-R* interval between vaccination and registration to the study, *PI mRNA* PI with BNT162b2 or mRNA-1273, *PI vector* PI with ChAdOx1, *PI mRNA/vector* PI with BNT162b2 or mRNA-1273 and ChAdOx1. Age in years, reference gender: male (divers: excluded due to small *n*), mSCQ: continuous variable, interval in days. Participants excluded from analysis: gender = divers: long-term survey: first vaccination: *n* = 2, second: *n* = 10, third: *n* = 2, follow-up survey: first/second vaccination: *n* = 10, third: *n* = 2; NA for “interval to 2^nd^ dose”: long-term survey: first vaccination: *n* = 2, second: *n* = 2; NA for “doctor’s consultation”: long-term survey: second vaccination: *n* = 2, follow-up survey: first/second vaccination: *n* = 2. *no events in this group

#### Long-term survey — second vaccination

No statistically significant associations were seen regarding the reporting of doctor’s consultations between the PI vaccination regimens. Regarding seeking hospital care both heterologous vaccination regimens were associated with lower odds compared to a homologous BNT162b2 regimen (ChAdOx1-BNT162b2: OR = 0.39, 95% CI [0.16, 0.91] and ChAdOx1-mRNA-1273: OR = 0.23, 95% CI [0.06, 0.71]). A higher mSCQ and higher vaccination-registration interval were associated with a higher risk for doctor’s consultation. Female gender was only associated with a higher risk regarding doctor’s consultation.

#### Long-term survey — third vaccination

No statistically significant associations between the vaccination regimens were seen regarding the reporting of doctor’s consultations and seeking hospital care 6 weeks after the third dose. A higher mSCQ was associated with a higher risk for both doctor’s consultations and seeking hospital care; a higher vaccination-registration interval only regarding doctor’s consultation.

#### Follow-up survey

The odds for reporting medical consultation in all vaccination regimens were comparable with the reference regimens with BNT162b2. A higher mSCQ and a longer interval between vaccination and registration were associated with a higher risk of reporting doctor’s consultations and seeking hospital care, respectively. Age, gender and the interval between the vaccinations had no significant influence (Table 4).

The additional analysis showed that a SARS-CoV-2 infection was associated with higher odds for reporting a doctor’s consultation. In the long-term survey after the second and third vaccination the odds for seeking hospital care were higher in participants who reported a SARS-CoV-2 infection after the last survey (Additional file 3: Table S2).

## Discussion

We performed an online survey to assess local and systemic reactogenicity, as well as utilisation of medical services after different COVID-19 vaccines and vaccination regimens. Most participants reported at least one reaction after a vaccination. Resulting of these, the majority reported no consequences. Most common consequences were medication intake and sick leave. A vaccination with mRNA-1273 was associated with higher odds for reporting local and systemic reactions than BNT162b2. Vector-based vaccines were associated with lower or equal odds for local reactions after the first and second dose and with a higher risk for reported systemic reactions after the first dose. A second dose of BNT162b2 in a heterologous regimen was associated with more frequent reporting of local and systemic reactions than in a homologous regimen. The regression analyses 124 days after the first and third dose, respectively, showed that the odds for reporting medical consultation in all vaccination regimens is comparable with the reference regimens with BNT162b2.

Reactogenicity after vaccinations differs widely among different observational studies. A study from Israel revealed a reactogenicity rate of 33–34% among persons older than 60 years and immunocompromised patients [29]. Self-reported local side effects were seen in 72%, 69% or 59% after a first or second dose of BNT162b2 or ChAdOx1 respectively [30]. Systemic side effects were reported less frequently (14%/22% or 34%). In a cohort study using patient-reported outcomes, 63% reported reactogenicity, 54% systemic reactions within seven days after the first vaccination [31]. A Japanese study analysing reactogenicity after BNT162b2 in healthcare workers showed a rate of 97% [32]. This heterogeneity can probably be attributed to several factors. As in our study, an association between reactogenicity and female gender and age has been shown before [13, 30, 31]. Therefore, the structure of the study population influences the rates. Amanzio et al. also suggested that a considerable part of reported adverse events might be attributed to nocebo effects [33]. They discuss that negative information, for example through the media, may further amplify the nocebo effect [34]. There was an enormous media attention on the COVID-19 vaccination, which possibly influenced the reporting of reactogenicity and adverse events. The magnitude of this influence may vary depending on the study population and the country.

Although reactogenicity rates differ between the studies, other research groups have shown comparable associations regarding the comparisons of vaccines and vaccination regimens as seen in our study. For example, Rolfes et al. reported that ChAdOx1 had the highest odds, followed by Ad26.COV2-S, mRNA-1273, and BNT126b2, for the outcome of systemic reactions after the first vaccination [31]. Higher odds for systemic reactions with ChAdOx1 than BNT162b2 were also shown by other studies [12, 30, 35]. In our results, we saw that a second ChAdOx1 vaccination was associated with less local reactions and comparable systemic reactions as compared to a second BNT162b2 vaccination.

Regarding differences between vaccination regimens after the second vaccination, Pfrommer et al. reported comparable results [12]. Heterologous vaccination regimens and the homologous mRNA-1273 regimen were associated with a higher reactogenicity than a homologous BNT162b2 regimen. A heterologous BNT162b2 vaccination regimen was associated with higher odds for systemic reactions. Other studies as well showed that the reactogenicity was higher after a heterologous vaccination regimen compared to a homologous one [36, 37].

Regression analysis showed that the reactogenicity or medical consultations after the third dose with BNT162b2 is hardly influenced by vaccination regimens administered for the PI. However, the odds for reporting systemic or local reactions were higher after mRNA-1273 compared to BNT162b2 (in a homologous regimen). Stuart and colleagues discussed that this could be due to the higher mRNA dosage of mRNA-1273 [11]. For the PI 100 µg mRNA is recommended for mRNA-1273 whereas for BNT162b2 only 30 µg [38, 39]. Given that for the mRNA-1273 booster vaccination a reduced dose is administered (50 µg) and that the association towards a higher reactogenicity with mRNA-1273 is less pronounced after the booster dose than after the first and second dose, this might support this hypothesis.

In other studies, local and systemic reactions after a vaccination were mainly classified as non-severe (98%) by the participants themselves [12] and only a minor percentage (5%) needed medical care for their symptoms [29]. In our study, if reactions led to consequences, those were mainly medication intake or sick leave. Analysing the differences between the vaccines and vaccination regimens, we saw only a few differences 6 or more weeks after the vaccination. The follow-up survey revealed no significant differences in odds for doctor’s consultations or seeking hospital care.

## Limitations and strengths

A large population was recruited in different settings (vaccination centres, primary care practices). Due to vaccination recommendations regarding age and gender, the results might not be generalisable for the German population. We therefore performed a logistic regression analysis. As it was possible to register for the study with a delay to the vaccination, it has to be considered, that our results might overestimate the reactogenicity and medical consultations. Participants might have registered due to symptoms occurring after the vaccination. Therefore, we included the variable “interval between vaccination and registration” in the logistic regression analyses which showed, that the length of the interval was associated with higher odds for reporting systemic reactions 14 days after all vaccinations, for reporting doctor’s consultation in the long-term and follow-up survey after the third vaccination and for reporting to seek hospital care in the follow-up survey after the third vaccination. When interpreting the results, one should consider that we analysed patient-reported outcomes. Regarding the reactogenicity queried in the short-term surveys, it can be assumed that local and systemic reactions are easily noticeable by the participants. As most of them do not lead to medical consultation, online surveys are an effective measure to capture them. However, when interpreting the results of the long-term and follow-up surveys, one should consider that the reports are not confirmed by physicians. In addition, we cannot make a statement on the causality. Very serious adverse events, or even death, cannot be detected by our method.

## Conclusions

Our analysis revealed differences in reactogenicity between the COVID-19 vaccines and vaccination regimens administered in Germany. Overall, participants with a BNT162b2 vaccination reported the lowest reactogenicity, especially in a homologous vaccination regimen. However, in all vaccination regimens reactogenicity within the first 2 weeks rarely led to medical consultations. Small differences in seeking any medical consultation 6 weeks after the vaccination diminished during the follow-up period. In the end, it seems that none of the vaccination regimes was associated with a higher risk for medical consultation.

## Supplementary Information

**Additional file 1.** STROBE checklist.

**Additional file 2.** Surveys.

**Additional file 3:Table S1.** Descriptive results of the short-term surveys. **Table S2.** Influence of SARS-CoV-2 infection on reporting outcomes. Logistic regression.

## Data Availability

Aggregated data that support the findings of this study are available from the corresponding author for researchers who provide a methodologically sound proposal after consent of the data protection supervisor.

## Abbreviations

CI: Confidence interval
CoVaKo: Corona Vakzin Konsortium
mSCQ-D: Modified German version of the Self-Administered Comorbidity Questionnaire
OR: Odds ratio
PI mRNA: PI with BNT162b2 or mRNA-1273
PI mRNA/vector: PI with BNT162b2 or mRNA-1273 and ChAdOx1
PI vector: PI with ChAdOx1
PI: Primary immunisation
REDCap: Research Electronic Data Capture
STROBE: Strengthening the Reporting of Observational studies in Epidemiology
WHO: World Health Organization
