# Supplementary material for: Reactogenicity and safety of COVID-19 primary immunisation and booster vaccination regimens: a comparative observational cohort study
Source: BMC Med. 2023 Jun 20;21:218. doi: 10.1186/s12916-023-02924-5 (PMC10283194; doi:10.1186/s12916-023-02924-5)
Supplement: Supplementary file 3 — Additional file 3:Table S1. Descriptive results of the short-term surveys. Table S2. Influence of SARS-CoV-2 infection on reporting outcomes. Logistic regression. [file 12916_2023_2924_MOESM3_ESM.pdf]

Additional File

Table S1: **Descriptive results of the short-term surveys**

	**After first vaccination Comparison of vaccines**				**After second vaccination Comparison of vaccination regimens**					**After third vaccination Comparison of vaccination regimens**					
	**mRNA**		**vector**		**hom. mRNA**		**hom. vector**	**het.**		**hom. PI mRNA**		**hom. PI vector**		**het. PI mRNA/vector**	
	BNT	1273	ChAd	Ad26	BNT-BNT	1273 1273	ChAd-ChAd	ChAd-BNT	ChAd-1273	BNT	1273	BNT	1273	BNT	1273
N	**2044**	**687**	**177**	**155**	**5391**	**1386**	**439**	**1098**	**820**	**884**	**782**	**84**	**59**	**204**	**229**
**Participants with COVID-19 infection after vaccination**															
%	0.3	0.1	0.0	0.0	0.2	0.2	0.0	0.1	0.0	1.7	0.6	0.0	0.0	0.0	0.0
NA	11	2	0	1	10	3	2	0	5	0	0	0	0	0	0
**Participants with at least one symptom (local, systemic or unsolicited)**															
%	70.3	83.0	85.3	80.6	72.9	91.1	56.2	82.5	90.7	71.4	80.8	59.5	71.2	75.5	70.7
N	1437	570	151	125	3931	1263	246	906	744	631	632	50	42	154	162
**Participants with at least one local reaction**															
%	57.5	73.9	54.2	47.7	55.8	78.7	32.6	61.6	77.2	57.4	69.2	47.6	52.5	56.9	58.1
n	1175	508	96	74	3007	1091	143	676	633	507	541	40	31	116	133
**Thereof percentage of participants who reported consequences**															
None	95.0	86.2	94.8	90.5	92.5	83.0	89.5	91.0	85.6	90.7	93.2	90.0	93.5	92.2	91.7
Medication intake	3.0	8.5	4.2	6.8	4.3	9.9	3.5	4.3	7.1	4.9	5.2	2.5	3.2	2.6	6.0
Sick leave	1.0	3.0	1.0	1.4	2.2	4.2	5.6	3.0	5.4	3.7	1.3	5.0	0.0	2.6	2.3
Ambulatory consultation	1.0	2.0	0.0	1.4	1.0	2.7	1.4	1.6	1.6	0.6	0.4	2.5	3.2	2.6	0.0
Hospital outpatient consultation	0.0	0.4	0.0	0.0	0.1	0.2	0.0	0.0	0.2	0.0	0.0	0.0	0.0	0.0	0.0
Hospitalisation	0.0	0.0	0.0	0.0	0.0	0.0	0.0	0.1	0.2	0.0	0.0	0.0	0.0	0.0	0.0
**Participants with at least one systemic reaction**															
%	48.5	59.2	81.9	77.4	61.8	85.1	48.5	76.0	85.5	58.1	69.3	42.9	61.0	63.7	57.2
n	992	407	145	120	3329	1180	213	834	701	514	542	36	36	130	131

**Thereof percentage of participants who reported consequences**															
None	71.3	67.1	46.2	40.8	58.6	40.1	58.2	44.7	36.4	54.1	54.2	52.8	66.7	50.0	55.7
Medication intake	19.5	21.6	37.9	33.3	25.5	36.4	26.8	34.3	36.9	28.0	31.4	16.7	27.8	33.1	25.2
Sick leave	4.5	5.4	11.0	23.3	11.4	18.5	11.7	15.0	20.3	13.4	11.1	25.0	5.6	11.5	16.0
Ambulatory consultation	3.8	5.9	4.8	2.5	3.8	4.7	2.8	4.4	5.1	3.9	3.1	5.6	0.0	5.4	2.3
Hospital outpatient consultation	0.6	0.0	0.0	0.0	0.4	0.3	0.5	1.1	1.0	0.4	0.0	0.0	0.0	0.0	0.8
Hospitalisation	0.3	0.0	0.0	0.0	0.2	0.0	0.0	0.5	0.3	0.2	0.2	0.0	0.0	0.0	0.0
**Participants with at least one unsolicited reaction**															
%	9.7	13.8	15.8	11.0	11.9	13.3	7.1	12.5	11.8	16.2	15.3	4.8	13.6	8.8	7.0
n	198	95	28	17	642	184	31	137	97	143	120	4	8	18	16
**Thereof percentage of participants who reported consequences**															
None	67.2	72.6	75.0	52.9	66.0	64.1	77.4	59.1	60.8	65.7	63.3	25.0	62.5	77.8	81.2
Medication intake	14.6	10.5	10.7	23.5	13.7	11.4	9.7	17.5	13.	12.6	15.8	25.0	12.5	0.0	6.2
Sick leave	6.1	2.1	3.6	23.5	5.6	8.7	3.2	7.3	9.3	7.0	10.0	0.0	12.5	11.1	6.2
Ambulatory consultation	11.1	14.7	7.1	0.0	12.3	14.1	9.7	12.4	13.4	13.3	9.2	50.0	12.5	5.6	6.2
Hospital outpatient consultation	0.5	0.0	3.6	0.0	1.4	1.6	0.0	2.2	2.1	0.7	0.8	0.0	0.0	5.6	0.0
Hospitalisation	0.5	0.0	0.0	0.0	0.9	0.9	0.0	1.5	1.0	0.7	0.8	0.0	0.0	0.0	0.0

Hom.: homologous vaccination regimen. Het: heterologous vaccination regimen. PI: primary immunization. BNT: BNT126b2. 1273: mRNA-1273. ChAd: ChAdOx1. Ad26: Ad26.COV2-S. PI mRNA = PI with BNT162b2 or mRNA-1273. PI vector: PI with ChAdOx1. PI mRNA/vector: PI with BNT162b2 or mRNA-1273 and ChAdOx1. Consequences named in hierarchical order (multiple choice was possible). The consequence perceived as most serious is reported (from no consequence to medication intake, sick leave, outpatient (practice) consultation, clinic (ambulant) consultation, and inpatient consultation).

Table S2: **Influence of SARS-CoV-2 infection on reporting outcomes. Logistic regression.**

a) Short-term survey after first vaccination (N=3060)

**Outcome: Local reactions**					**Outcome: Systemic reactions**			
	OR	95%-CI		p	OR	95%-CI		p
Intercept	2.9577	2.2780	3.8475	0.00000	1.8049	1.3952	2.3369	0.00001
1273	2.0550	1.6872	2.5116	0.00000	1.4865	1.2401	1.7838	0.00002
ChAd	1.1051	0.7970	1.5360	0.55006	6.3976	4.3069	9.7755	0.00000
Ad26	0.6803	0.4836	0.9557	0.02639	4.2239	2.8664	6.3743	0.00000
Age	0.9710	0.9656	0.9763	0.00000	0.9716	0.9663	0.9769	0.00000
Female	2.0996	1.8001	2.4501	0.00000	1.7987	1.5429	2.0980	0.00000
mSCQ	1.0432	0.9917	1.0979	0.10290	1.1873	1.1270	1.2527	0.00000
Interval vaccination-registration	1.0125	0.9891	1.0366	0.30009	1.0290	1.0053	1.0535	0.01685
SARS-CoV-2 infection since vaccination	0.2652	0.0518	1.1238	0.08027	1.8033	0.4392	9.2260	0.43262

b) Long-term survey after first vaccination (N=3027)

**Outcome: Doctor’s consultation**					**Outcome: Hospital care**			
	OR	95%-CI		p	OR	95%-CI		p
Intercept	0.1032	0.0693	0.1524	0.00000	0.0136	0.0047	0.0368	0.00000
1273	1.1672	0.8943	1.5134	0.24891	1.0504	0.4801	2.1194	0.89554
ChAd	1.6712	1.0837	2.5076	0.01609	2.1914	0.7295	5.3729	0.11577
Ad26	1.6889	1.0512	2.6242	0.02420	1.5466	0.3633	4.5160	0.48244
Age	0.9916	0.9840	0.9992	0.03027	0.9858	0.9659	1.0056	0.16124
Female	1.6353	1.3050	2.0569	0.00002	1.4477	0.8080	2.6757	0.22294
mSCQ	1.2739	1.2048	1.3476	0.00000	1.2625	1.1220	1.4037	0.00004
Interval vaccination-registration	1.0060	0.9981	1.0136	0.12820	1.0177	0.9989	1.0354	0.05346
SARS-CoV-2 infection since last survey	7.7373	2.5949	23.0726	0.00018	5.3177	0.2865	28.5068	0.11486

c) Short-term survey after second vaccination (N=9104)

**Outcome: Local reactions**					**Outcome: Systemic reactions**			
	OR	95%-CI		p	OR	95%-CI		p
Intercept	3.4808	2.6673	4.5488	0.00000	7.0459	5.2756	9.4407	0.00000
1273,1273	3.0953	2.6804	3.5837	0.00000	3.8259	3.2550	4.5167	0.00000
ChAd,ChAd	0.5671	0.4198	0.7645	0.00021	1.1316	0.8311	1.5439	0.43387
ChAd,BNT	1.4891	1.1852	1.8733	0.00065	2.9743	2.3062	3.8530	0.00000
ChAd,1273	3.1589	2.3878	4.1925	0.00000	5.5164	4.0213	7.6163	0.00000
Age	0.9702	0.9670	0.9734	0.00000	0.9665	0.9631	0.9699	0.00000
Female	2.1073	1.9238	2.3087	0.00000	1.9836	1.8021	2.1837	0.00000
mSCQ	1.0889	1.0584	1.1205	0.00000	1.1243	1.0906	1.1596	0.00000
Interval between vaccinations	0.9975	0.9917	1.0033	0.39120	0.9912	0.9849	0.9975	0.00636
Interval vaccination-registration	1.0070	1.0046	1.0094	0.00000	1.0081	1.0056	1.0106	0.00000
SARS-CoV-2 infection since vaccination	0.5656	0.1942	1.5511	0.27449	0.6699	0.2406	1.8804	0.43772

d) Long-term survey after second vaccination (N=9328)

**Outcome: Doctor's consultation**					**Outcome: Hospital care**			
	OR	95%-CI		p	OR	95%-CI		p
Intercept	0.0885	0.0619	0.1256	0.00000	0.0098	0.0034	0.0268	0.00000
1273,1273	1.1506	0.9736	1.3556	0.09642	0.8786	0.5246	1.4001	0.60346
ChAd,ChAd	0.7278	0.4825	1.0840	0.12359	0.9610	0.3194	2.6320	0.94108
ChAd,BNT	0.8543	0.6417	1.1301	0.27523	0.4654	0.1874	1.0601	0.08292
ChAd,1273	0.8025	0.5660	1.1296	0.21193	0.2857	0.0815	0.8640	0.03650
Age	0.9984	0.9942	1.0025	0.44075	1.0039	0.9923	1.0156	0.51484
Female	1.6351	1.4456	1.8519	0.00000	0.8944	0.6420	1.2511	0.51117
mSCQ	1.2375	1.2005	1.2757	0.00000	1.2215	1.1397	1.3028	0.00000
Interval between vaccinations	1.0052	0.9976	1.0130	0.18285	1.0059	0.9838	1.0299	0.61639
Interval vaccination-registration	1.0102	1.0070	1.0134	0.00000	1.0166	1.0073	1.0266	0.00067
SARS-CoV-2 infection since last survey	8.7941	4.4012	18.0569	0.00000	5.3004	1.2464	15.3881	0.00709

e) Follow-up survey after first/second vaccination (N=9591)

**Outcome: Doctor's consultation**					**Outcome: Hospital care**			
	OR	95%-CI		p	OR	95%-CI		p
Intercept	1.2434	0.7455	2.0932	0.40703	0.0649	0.0187	0.1890	0.00000
1273,1273	1.0941	0.9397	1.2709	0.24305	0.9907	0.6672	1.4303	0.96155
ChAd,ChAd	0.8647	0.6078	1.2197	0.41282	0.9294	0.3814	2.1020	0.86622
ChAd,BNT	0.8823	0.6837	1.1337	0.33173	0.5448	0.2663	1.0558	0.08349
ChAd,1273	0.7878	0.5723	1.0787	0.14011	0.4239	0.1622	1.0181	0.06623
Age	0.9975	0.9938	1.0012	0.18739	0.9972	0.9879	1.0066	0.55879
Female	1.3714	1.2300	1.5301	0.00000	0.8546	0.6532	1.1213	0.25366
mSCQ	1.2630	1.2277	1.2996	0.00000	1.2094	1.1410	1.2784	0.00000
Interval between vaccinations	0.9978	0.9913	1.0045	0.51493	0.9979	0.9810	1.0158	0.80990
Interval vaccination-registration	1.0085	1.0063	1.0108	0.00000	1.0156	1.0102	1.0209	0.00000
SARS-CoV-2 infection since last survey	6.5338	4.1758	10.3572	0.00000	2.2848	0.7866	5.2681	0.08206

f) Short-term survey after third vaccination (N=2241)

**Outcome: Local reactions**					**Outcome: Systemic reaction**			
	OR	95%-CI		p	OR	95%-CI		p
Intercept	2.3197	1.7154	3.1430	0.00000	3.3265	2.4481	4.5336	0.00000
PI mRNA + mRNA-1273	2.3341	1.8740	2.9147	0.00000	2.4870	1.9911	3.1154	0.00000
PI vector + BNT162b2	1.0768	0.6702	1.7286	0.75884	0.8789	0.5416	1.4179	0.59817
PI vector + mRNA-1273	1.2670	0.7318	2.2040	0.39809	1.8848	1.0787	3.3550	0.02785
PI mRNA/vector + BNT162b2	1.0727	0.7816	1.4765	0.66511	1.4180	1.0241	1.9754	0.03698
PI mRNA/vector + mRNA-1273	1.8178	1.3201	2.5143	0.00027	1.8823	1.3640	2.6093	0.00013
Age	0.9721	0.9657	0.9786	0.00000	0.9637	0.9571	0.9702	0.00000
Female	1.9851	1.6579	2.3787	0.00000	1.6915	1.4097	2.0307	0.00000
mSCQ	1.1035	1.0390	1.1738	0.00154	1.1283	1.0607	1.2026	0.00016
Interval vaccination-registration	1.0090	0.9805	1.0386	0.54264	1.0496	1.0188	1.0821	0.00162
SARS-CoV-2 infection since vaccination	1.0414	0.4099	2.8655	0.93374	1.5802	0.5863	5.0139	0.39349

g) Long-term survey after third vaccination (N=2371)

**Outcome: Doctor's consultation**					**Outcome: Hospital care**			
	OR	95%-CI		p	OR	95%-CI		p
Intercept	0.1438	0.0961	0.2131	0.00000	0.0076	0.0022	0.2280000	0.00000
PI mRNA + mRNA-1273	0.9551	0.7313	1.2466	0.73549	1.1159	0.5368	2.336000	0.76808
PI vector + BNT162b2	1.2638	0.7058	2.1694	0.41152	2.6940	0.8221	7.577300	0.07458
PI vector + mRNA-1273	0.4508	0.1642	1.0338	0.08472	NA*	NA*	NA*	NA*
PI mRNA/vector + BNT162b2	0.7717	0.4963	1.1674	0.23356	1.1094	0.3111	3.132700	0.85639
PI mRNA/vector + mRNA-1273	0.9210	0.5930	1.3998	0.70666	0.4664	0.0717	1.741400	0.32349
Age	0.9961	0.9878	1.0043	0.35039	1.0099	0.9877	1.032300	0.37806
Female	1.1808	0.9360	1.4933	0.16275	0.7099	0.3760	1.339800	0.28726
mSCQ	1.2875	1.2095	1.3710	0.00000	1.2650	1.0863	1.449600	0.00128
Interval vaccination-registration	1.0252	1.0076	1.0423	0.00386	1.0399	0.9969	1.077100	0.04460
SARS-CoV-2 infection since last survey	3.2445	1.7012	5.9665	0.00021	5.3910	1.2476	16.20680	0.00767

h) Follow-up survey after third vaccination (N=2304)

**Outcome: Doctor's consultation**					**Outcome: Hospital care**			
	OR	95%-CI		p	OR	95%-CI		p
Intercept	0.2560	0.1816	0.3592	0.00000	0.0157	0.0058	0.0390	0.00000
PI mRNA + mRNA-1273	1.0359	0.8345	1.2858	0.74912	0.7556	0.4060	1.3832	0.36701
PI vector + BNT162b2	0.9235	0.5653	1.4766	0.74459	1.1565	0.3283	3.1544	0.79622
PI vector + mRNA-1273	0.7712	0.4075	1.3924	0.40446	0.9869	0.1550	3.4858	0.98602
PI mRNA/vector + BNT162b2	0.8212	0.5791	1.1514	0.26039	1.5689	0.6816	3.3031	0.25756
PI mRNA/vector + mRNA-1273	0.8449	0.5985	1.1823	0.33126	0.6284	0.2066	1.5716	0.35949
Age	1.0015	0.9947	1.0082	0.66513	1.0132	0.9957	1.0308	0.13796
Female	1.1952	0.9910	1.4429	0.06269	0.8819	0.5308	1.4772	0.62876
mSCQ	1.1949	1.1294	1.2649	0.00000	1.1375	0.9960	1.2796	0.04233
Interval vaccination-registration	1.0140	1.0062	1.0222	0.00050	1.0137	0.9984	1.0261	0.04535
SARS-CoV-2 infection since last survey	1.7509	1.4212	2.1557	0.00000	0.6514	0.3076	1.2488	0.22556

Note: Hom.: homologous vaccination regimen. Het: heterologous vaccination regimen. PI: primary immunization. BNT: BNT126b2. 1273: mRNA-1273. ChAd: ChAdOx1. Ad26: Ad26.COV2-S. PI mRNA = PI with BNT162b2 or mRNA-1273. PI vector: PI with ChAdOx1. PI mRNA/vector: PI with BNT162b2 or mRNA-1273 and ChAdOx1. mSCQ: modified German version of the Self-Administered Comorbidity Questionnaire. Reference vaccine or vaccination regimen: BNT162b2 or BNT162b2 + BNT162b2 or mRNA primary immunisation + BNT162b2. Age in years, reference gender: male (divers: excluded due to small n), mSCQ: continuous variable, interval in days. *no events in this group
